# Cross-Tolerance and Autoimmunity as Missing Links in Abiotic and Biotic Stress Responses in Plants: A Perspective toward Secondary Metabolic Engineering

**DOI:** 10.3390/ijms222111945

**Published:** 2021-11-04

**Authors:** Lakshmipriya Perincherry, Łukasz Stępień, Soniya Eppurathu Vasudevan

**Affiliations:** 1 Department of Plant-Pathogen Interaction, Institute of Plant Genetics, Polish Academy of Sciences, Strzeszyńska 34, 60-479 Poznań, Poland; lste@igr.poznan.pl; 2 Department of Transdisciplinary Biology, Rajiv Gandhi Centre for Biotechnology, Thycaud, Thiruvananthapuram 695014, Kerala, India; evsoniya@rgcb.res.in

**Keywords:** autoimmunity, stress tolerance, metabolic engineering

## Abstract

Plants employ a diversified array of defense activities when they encounter stress. Continuous activation of defense pathways that were induced by mutation or altered expression of disease resistance genes and mRNA surveillance mechanisms develop abnormal phenotypes. These plants show continuous defense genes’ expression, reduced growth, and also manifest tissue damage by apoptosis. These macroscopic abrasions appear even in the absence of the pathogen and can be attributed to a condition known as autoimmunity. The question is whether it is possible to develop an autoimmune mutant that does not fetch yield and growth penalty and provides enhanced protection against various biotic and abiotic stresses via secondary metabolic pathways’ engineering. This review is a discussion about the common stress-fighting mechanisms, how the concept of cross-tolerance instigates propitious or protective autoimmunity, and how it can be achieved by engineering secondary metabolic pathways.

## 1. Introduction

Plants face many constraints in the continuously changing environment and have evolutionarily gained the defensive mechanisms that are inevitable for their survival. Numerous efforts have been taken for a better understanding of plant adaptive responses to improve agricultural productivity and quality. When plants encounter particular stress, numerous genes are induced, which results in elevated levels of diverse secondary metabolites and proteins that would help plants to cope with the stress [1]. These metabolites aid hypersensitive cell death, signal transduction, and downstream defense gene activation, e.g., the pathogenesis-related (PR) genes, enzymes for phytoalexin biosynthesis, oxidative stress, tissue repair, cell wall lignification, and several other cellular processes [2]. It has been postulated that a cross-talk exists between the plant physiological, cellular, and molecular responses, through which the plant reacts via phytohormones, transcription factors, kinase cascades, and reactive oxygen species [3,4,5]. In certain conditions, the synergistic or antagonistic cross-talk can lead to another phenomenon called cross-tolerance, which yields supplementary resistance against pathogens [6]. The continuously evolving pathogen races are recognized by intracellular immune receptors belonging to the family of polymorphic nucleotide-binding oligomerization, domain-containing leucine-rich repeat (NB-LRR) proteins that trigger the typical, programmed cell death as a consequence of hypersensitive response [7]. Plants that are exposed to one stress can acclimatize and form a stress memory, which also helps them to impede the damage caused by other stresses [8]. On the other hand, the loss of function, consecutive overexpression of certain genes, or inappropriate activation of respective proteins associated with plant immunity promotes negative regulators, which induce macroscopic lesions, resembling symptoms of the disease in the absence of pathogens. Such mutants can thereby be called autoimmune phenotypes [9]. Numerous studies were carried out to unveil the mechanism of cross-tolerance and induced autoimmunity in various plants [10,11]. Metabolic engineering and transcription-factor-based technologies are apparently suitable and promising for the next generation of multiple-stress-tolerant crop plants [12,13]. Several candidate genes encoding the common stress-interactive elements were identified, and their functional analysis was carried out using mutation studies (see Glossary). Here, we discuss the role of various stress-signaling factors, and how they contribute to the concept of cross-tolerance and plant autoimmunity. We also emphasize the engineering of metabolic and signaling pathways for creating multiple-stress-resistant plants.

## 2. Common Abiotic and Biotic Stress Tolerance Mechanisms

### 2.1. Abiotic Stress Tolerance

A certain physiological effect that disturbs the homeostasis and overall cellular metabolism of a living organism is called stress. Abiotic causes of stress, such as drought, salinity, heavy metals, low and high temperature, can disrupt the physical and biochemical homeostasis of plants and can thereby induce variation in the cellular aqueous and ionic balances [14]. As a result, hundreds of genes become transcribed, and their products undergo translational modifications [15,16]. Osmoprotectants and reactive oxygen species (ROS) scavengers are crucial among them. Glycine betaine, which is a quaternary ammonium compound, occurs abundantly during dehydration stress [17,18,19] and helps in maintaining the photosynthetic efficiency of the thylakoid membrane. The amino acid proline plays a similar role, as it helps to maintain the osmotic balance and stabilization of membranes and proteins, to scavenge the ROS, and to maintain a redox potential during stressful conditions [18]. ROS scavenging, on the other hand, is accomplished by enzymatic antioxidant systems, which include superoxide dismutase, catalase, ascorbate peroxidase, glutathione reductase, mono-dehydroascorbate reductase, dehydroascorbate reductase, glutathione peroxidase, guaiacol peroxidase, glutathione-*S*-transferase and non-enzymatic antioxidant systems, such as ascorbic acid, glutathione, tocopherol, carotenoids, and flavonoids [20,21]. Moreover, a multitude of supplemental proteins and transcription factors are also upregulated during all types of abiotic stress. When the functionality of the cDNA′s in a diverse combination of stress is compared, the functional gene categories show an analogous pattern, and they also exhibit a higher expression under a common stress response machinery. For example, cDNA libraries of barley plants show significant functional similarities under drought and saline conditions. Proteins such as late-embryogenesis abundant (LEA), metallothionein-like (ML), jasmonate responsive, and ABA-responsive, are remarkably upregulated under drought stress, whereas ubiquitin-related transcripts are upregulated in salinity stress, along with LEA and ML [22]. Apart from this, transcripts encoding jasmonate biosynthesis and jasmonate responsive proteins were derived by cDNA microarray from dehydration-shocked barley leaf tissues [23]. There is a strong relationship between the expression profiles of cold-, drought- and high-salinity-stress-induced plants. Similarly, transcripts that code for various transcription factors, influencing osmoprotective protein synthesis, detoxification, lipid transfer, water channeling, fatty acid metabolism, hormone biosynthesis, heat shock proteins, etc. were commonly expressed in Arabidopsis during drought, cold, and high-salinity stress. It is also known that polyamines such as spermine and putrescine play remarkable roles in the abiotic stress tolerance in plants and are highly expressed during drought [24], saline [25], and chilling stress [26]. The exogenous application of polyamines has been proven to enhance the endogenous polyamine levels and are associated with maintenance of membrane integrity, accumulation of osmolytes, reduction in ROS levels, lower levels of Na^+^ and Cl^−^ in the organelles, and reduced negative impact on photosynthetic machinery, compared with the non-treated plants [27]. It was also suggested that polyamines prevent lipid peroxidation and quenching of free radicals by binding to cellular/organelle membranes [28].

Although several routes of chemical defense against abiotic stresses exist, the physical barrier created by the cuticle functions as the outermost shield. Cutin, being a macromolecular polyester consisting of C16/C18 fatty acids, is synthesized de novo from acetyl coenzyme A in plastids and oxygenated in the endoplasmic reticulum (ER) to serve as cutin precursor or is modified to become cuticle wax [29]. These fatty acids are present in the cuticle but are also key components of biological membranes and come into play in maintaining membrane fluidity. Membrane remodeling is of great importance, as cold stress rigidifies the membrane, whereas heat stress fluidizes it. Both can lead to membrane dysfunction and ion/cellular component leakage. The polyunsaturated fatty acids, upon reaction with lipase, release raw materials of several other bioactive compounds such as oxylipins and jasmonates, which are implicated for defense against other types of stress. Additionally, jasmonates are also well studied for their ability to provide resistance against salt stress and UV stress [30,31].

### 2.2. Biotic Stress Tolerance

Biotic stress in plants is caused by living organisms such as bacteria, viruses, fungi, nematodes, insects, weeds, or herbivorous animals. The constant evolution of molecular studies on plant immunity and pathogen infection strategies reveals an integrated picture of the plant–pathogen interaction. Plants recognize and exhibit defense responses against various pathogens through approaches involving the recognition of both conserved and variable pathogen elicitors, as well as secreted virulence effectors of pathogens [32]. Plants bring forth defense responses upon the perception of a pathogen, which comprises pathogen-associated molecular patterns (PAMPs)—pathogen-triggered immunity (PTI) and effector-triggered immunity (ETI) [33]. Upon perception of the pathogen effector molecules by R genes’ products, various processes occur in the cell, such as calcium ion influx, production of reactive oxygen species, activation of protein kinases, etc. [34]. Later, transcripts associated with signaling molecules or molecules analogous to defense execution and metabolism are accumulated, which leads to amassing of downstream signaling molecules, such as salicylic acid (SA), jasmonate (JA), ethylene (ET), and, finally, accomplishing the objective of defense signal transduction through cell wall strengthening, lignification, phytoalexin synthesis, and programmed cell death [35]. The defense against wounding stress by insects/herbivores also involves jasmonate signaling, where biosynthesis of leaf endoplasmic reticulum (L-ER) bodies, playing major roles in wounding resistance, can be JA dependent/independent. The L-ER synthesizes two different kinds of β-glucosidases—BGLU18 and PYK10. The expression of *BGLU18* depends on JA signaling, and *PYK10* is JA independent. Plants constantly adjust their physiology and metabolism for recognizing, integrating, and defending various stresses, which is facilitated by the cross-tolerance phenomenon that makes the plant immune against continuously changing environmental variables [5]. Majority of the plant defense execution involves a range of secondary metabolites that are the products of metabolic pathways such as phenylpropanoid pathway, mevalonic/non-mevalonic acid pathway (isopentanoyl pathway) [36], and polyketide pathway [37]. In plants, type III polyketide synthases are involved in catalyzing sequential decarboxylative Claisen condensations of malonyl CoA with other CoA-linked starter molecules, that are metabolites of supplementary pathways, to produce important secondary metabolites active in plant immune responses [38].

## 3. The Concepts of Cross-Talk and Cross-Tolerance

**Figure 1:**
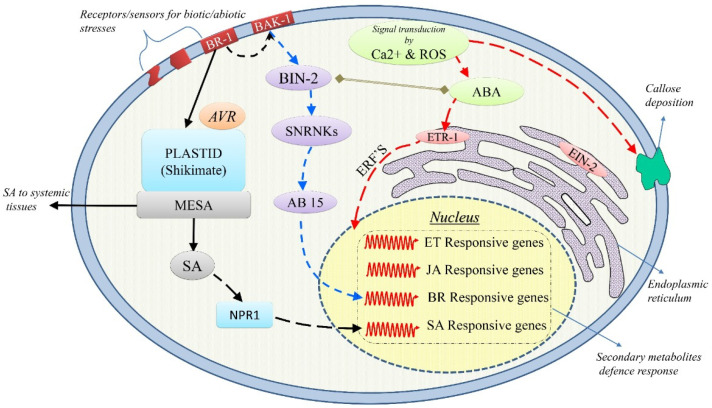
Possible role of plant hormones in stress cross-tolerance. The biotic and abiotic stress factors are first sensed by the plant cell membrane and are transduced to specific downstream signaling pathways. Reactive oxygen species (ROS) and Ca^2+^ play major roles as messenger molecules that initiate ABA-mediated cell signaling. It was discovered that ethylene-insensitive 2 (EIN2) and ethylene-responsive genes (ETR) of Arabidopsis are involved in ABA responses. They activate the ethylene-responsive transcription factors (ERFs), which are key regulatory hubs that integrate ethylene, ABA, and jasmonate signaling and thereby their corresponding responsive gene transcription. Brassinosteroids are perceived by the extracellular domain of Brassinosteroid-insensitive 1 (BRI1)—a leucine-rich repeat kinase located in the plasma membrane. Upon perception, the intercellular serine–threonine kinase domain of BRI1 becomes phosphorylated and dissociates to form a complex with a second receptor kinase BRI1-associated receptor kinase BAK1. This transduces the signal by inactivating another kinase Brassinosteroid-insensitive 2 (BIN2), thus activating Abscisic acid-insensitive 5 (ABI5), a bzip transcription factor that regulates the transcription of ABA- and BR-responsive genes. Increased biosynthesis of SA occurs upon pathogen attack by effector perception via ICS/PAL pathway in plastids. Conversion of SA to methyl salicylate (MeSA) is catalyzed by SA methyltransferase (SAMT). MeSA diffuses into the cytoplasm, where it is converted back to SA and is distributed to the systemic tissues. Increased levels of SA in the cytoplasm lead to the disruption of oligomeric NPR1 into its monomers, migrating to the nucleus to activate transcription of SA-responsive defense genes including PR genes.

Throughout their lifetime, plants stumble across various environmental hazards including insect and herbivore attacks, pathogens, and other physical factors, such as temperature, salinity, drought, etc. They react moderately by activating the primary immune response that has evolved to recognize and counteract as a highly specific defense against the stress encountered [39]. A plant that successfully defends against one stress has the possibility to become more resistant to many other stresses when it is exposed to multiple types of stress. This phenomenon is called cross-tolerance, and it shows that plants have evolved powerful defense regulatory pathways that allow them to quickly adapt to the changing environment [6]. Cross-tolerance is achieved by the cross-talk between plant defense signaling and metabolic pathways where these interactions can be mutually antagonistic or synergistic, leading to negative or positive functional outturn [40]. Cross-talk minimizes the energy spent and helps the plant to create a flexible signaling network that allows the plant to fine-tune its defense response to the invaders encountered [2,40,41]. This mechanism is extremely important because plants can be bred selectively, and their genes can be manipulated to render them tolerant to multiple stresses. Salicylic acid, abscisic acid, jasmonic acid, and ethylene are the most studied mediators and were proposed as the key players in stress tolerance in plants (Figure 1) [32,42,43,44,45]. Several other plant growth hormones are also important in cross-tolerance signaling mechanisms. Brassinosteroids—the endogenous plant growth hormones—upregulate the antioxidant levels by accumulating apoplastic H_2_O_2_ and thereby provide stress tolerance [46]. The cell surface receptor kinase brassinosteroid insensitive 1 (BRI1) perceives brassinosteroids, which triggers a signal cascade in the cytoplasm that leads to the transcription of BR-responsive genes [47]. They have multifaceted roles in pathogen interaction, PAMP reception, activation of stress response genes, fine-tuning of oxidative metabolism, and production of secondary metabolites, thus contributing to innate immunity, PTI, and cell death [48]. The classic ethylene-mediated signal transduction is triggered from the membrane receptors (*ETR* genes) and constitutive triple response—MKKK (CTR1) modulation—to regulate the activity of several genes [49]. In the presence of ethylene, the ethylene insensitive-2 (EIN2) domain is cleaved, and it is transported into the nucleus to repress EBF and activate EIN3/EIL, which, in turn, activates several transcription factors that interact with other phytohormones, such as JA and SA, regulating them positively or negatively [50]. The ethylene-responsive factors ERF1, ERF2, and ERF5 regulate the expression of JA-responsive gene plant defensin 1.2 (*PDF1.2*) [51]. Similarly, the jasmonate insensitive/MYC2 (JIN/MYC2) is the transcription factor that plays an important role in JA-responsive gene expression [52]. The F-box protein and component of SCF E3 ubiquitin ligase, coronatine insensitive 1 (COL1) also has an important role in JA signaling, along with JAZ and MYC. They constitute the major signaling intersection of various stress-responsive pathways. The JAZ/MYC or other JAZ/TF elevates the concentration of secondary metabolites that initiate defense response/hypersensitive response against the pathogen interaction [53]. Generally, JA is involved mainly in regulating defense responses against necrotrophic pathogens, whereas SA-mediated defense activation is for broad-spectrum resistance against both biotrophic and hemibiotrophic pathogens [54]. SA is derived from the shikimate–phenylpropanoid pathway in the chloroplasts and is transported to the cytosol under tight signaling control. The consecutive accumulation of SA in the cytosol can lead to reduced fitness of the plant. It has been postulated that the biosynthesis of SA at the site of infection can induce gene expression in the distal part of the plant. The hormone induces the expression of PR genes, thus initiating systemic acquired resistance [55]. There is a significant overlap between these secondary metabolic pathways [56] along with a common signal (such as ROS) that triggers downstream signaling pathways [57], which renders the plants tolerant against multiple stresses. There is a remarkable coincidence of many defense-related and stress-responsive genes that are routinely taking part in the reaction to multiple stresses. Apart from these, several transcription factors are also upregulated, such as ethylene-responsive factor 1 [58], NAC6 [59], ABA-responsive element (ABRE) [60], WRKY transcriptional factors [61], etc. It is also evident that flavonoids and carotenoids play major roles in protecting the plants from oxidative and pathogen stresses [20,21].

## 4. Expediency in Plant Autoimmunity: The Propitiously Misdirected Process

**Figure 2:**
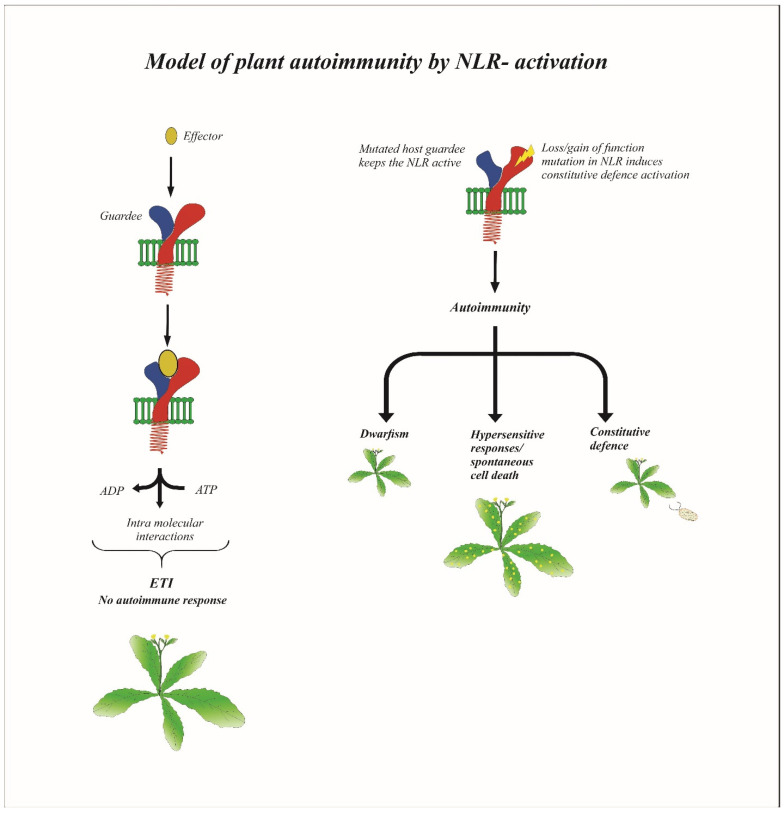
Model of plant autoimmunity by NLR-activation. Modification of guardee protein by the effector is perceived by NLR receptor thereby activating a strong immune response termed as effector-triggered immunity (ETI). Induced mutations/knockouts of host guardee or loss/gain of function mutations in NLR proteins initiates constitutive downstream defense signaling, resulting in autoimmunity characterized by dwarfism, hypersensitive responses, and increased defense against pathogens.

Plant immune systems are under strict control to avoid continuous activation of defense response without the presence of pathogens, and when this control is lost, it leads to autoimmunity, which may be at the expense of plants’ growth and development. Normally, plant autoimmunity is induced by certain mutations in genes encoding nucleotide-binding leucine-rich repeat (NLR) immune receptors, where there is continuous activation of the defense genes and related proteins (Figure 2) [62]. In some hybrid plants, autoimmunity can be naturally developed by the heteromeric association of autoimmune risk alleles of two NLRs. In Arabidopsis, the P-loop and Toll interleukin (TIR) domain-mediated heteromeric association of two different NLR locus variants—DM1 and DM2d—leads to the activation of the DM1 complex, which can activate immune signaling in the absence of non-self-recognition [63]. Continuous activation of defense response can also occur when there is an aberration in the RNA surveillance mechanism that degrades mutated mRNAs. One of such mechanisms is the nonsense-mediated mRNA decay (NMD) that regulates the level of functional proteins expressed during defense responses in plants. The functionally marred NMD proteins, such as SMG7, UPF1, or UPF3, result in constitutive activation of NLR and thereby induce autoimmune responses [64]. The typical phenotypes of autoimmune mutants include dwarfism, elevated salicylic acid levels, constitutive expression of defense genes and enhanced disease resistance to pathogens, and in some cases also spontaneous lesion formation [65]. On the other hand, an aberration in the NLR protein regulation and constitutive expression of R genes can also lead to non-pathogen-induced defense activation, thus leading to autoimmunity [66,67]. To summarize, it can be clearly stated that the constitutive expression of any distinct gene that continuously activates the immune response proceeds to autoimmunity; this misdirected process can be propitious if it does not compromise the growth, reproduction, and yield of the plant.

Apart from mutations and aberrations in NB-LRR, autoimmunity can also be developed by disruption or constitutive expression of genes involved in various cellular processes. Studies on induced constitutive gene expression were initiated by [68]. Initially, the studies were based on fungal cell wall-degrading enzymes such as chitinase and glucanase. Transgenic tobacco seedlings constitutively expressing a bean chitinase gene under the control of the cauliflower mosaic virus 35S promoter showed increased tolerance to the fungal pathogen *Rhizoctonia solani* present in the soil and delayed appearance of disease symptoms. Studies are being conducted to evaluate the resistance offered by altered or overexpressed genes involved in cellular signaling, whose continuous and elevated expression leads to defense activation. One of the examples is calcium-dependent protein kinase (CDPK). CDPKs are cellular secondary messengers and principal mediators of downstream responses in plant immune and stress signaling [69]. The ginger *CDPK1* gene, overexpressed in tobacco, was found to be rendering salinity and drought stress tolerance [70]. Apart from the common autoimmune phenotypes, the plants showed a high percentage of seed germination, higher relative water content, expression of stress-responsive genes, higher leaf chlorophyll content, increased photosynthetic efficiency, and other photosynthetic parameters. Similarly, potato aspartic proteases under constitutive expression in *A. thaliana* forged the plant cytotoxic to the fungal pathogen *Botrytis cinerea*, along with rendering improved growth and development [71]. The *PDF1.2* gene involved in the jasmonate signaling pathway was found to be induced after infection with the pathogen. On the other hand, it becomes responsible for the activation of salicylic acid-associated genes such as *PR1* even in the absence of the pathogen. The SA-dependent activation of *PR* genes involves NPR proteins that both positively and negatively regulate their expression. In Arabidopsis, the NPR1 is a positive regulator of SAR, which enhances SA-induced *PR* gene expression. Elucidating the function of NPR3 and NPR4 by mutation studies showed constitutive expression of *PR1*, which induced autoimmune responses [72]. The single and double mutants exhibited enhanced disease resistance to virulent bacterial and oomycete pathogens. A ubiquitin protein (PUB13) that selectively degrades proteins that are intertwined and affect plant processes involving growth, development, defense, and other cellular process mediates SA-mediated defense signaling and flowering time in Arabidopsis. The *PUB13*-disrupted plants resulted in spontaneous cell death, the accumulation of hydrogen peroxide and salicylic acid (SA), and elevated resistance to biotrophic pathogens but increased susceptibility to necrotrophic pathogens. It is evident that there is a possibility to develop autoimmune genotypes, which show constitutive defense response without growth and yield penalty.

## 5. Targeting Secondary Metabolite Biosynthetic Pathways for Multiple-Stress Resilience and Induced Autoimmunity

Apart from the continuous activation and impaired regulation of NLR proteins, we propose that certain mutations in the secondary metabolic pathway genes can induce autoimmunity and can also help plants to be resilient to multiple types of stress. The list of autoimmune phenotypes with a continuous expression of resistance-related genes is given in Table 1. Generally, an enormous number of secondary metabolites are synthesized via the phenylpropanoid pathway by using phenylalanine—one of the final products of the shikimate pathway, and they have a wide variety of functions, both as structural and signaling molecules. The initial substrate phenylalanine undergoes deamination by the enzyme phenylalanine ammonia lyase (PAL) and is converted to cinnamate [73], followed by generation of coumarate and other acids with a phenylpropane unit through hydroxylation and continual methylation reaction. Reduction in the CoA-activated carboxyl groups of these acids results in the corresponding aldehydes and alcohols. The hydroxycinnamyl alcohols called monolignols are the starting compounds for the biosynthesis of lignin [74], which is the second most abundant biopolymer that provides structural and vascular integrity to plants, as well as their resistance to pathogens.

Flavonoids are proven to be among the protecting agents in plants during high light and UV stresses [75]. The flavonoid biosynthetic pathway gives rise to a wide array of compounds that are attributed to various biological activities and are derived from the acetyl–CoA, malonyl–CoA, and phenylpropanoid pathways. They are a chemically diverse and biologically important group of secondary metabolites that are further subdivided into anthocyanidins, flavonols, flavones, chalcones, dihydrochalcones, and dihydroflavonols [76]. Anthocyanidins are the sugar-free counterparts of the plant pigment anthocyanins. These pigments deliver UV protection to plants during light and extreme temperature stresses [77]. Certain antioxidant enzymes are inactivated during severe stress, simultaneously the synthesis of some specific flavonols becomes upregulated. They are also involved in the reduction in ROS species, which, in turn, helps the plants in the suppression of oxygen radicals, inhibition of ROS-generating enzymes such as lipoxygenase, cyclooxygenase, monooxygenase, etc. [78].

Even though the functions of many flavonoids are yet to be revealed, there is increasing evidence that they are inevitable factors for eliciting defense response in many plant species. Phenolic acids such as hydroxyl benzoic acid and hydroxyl cinnamic acid produced from the intermediate steps of the phenylpropanoid pathway are involved in attracting the beneficial microorganisms and establishing a symbiotic relationship with the plant roots [79]. It is possible to engineer the metabolic pathways of therapeutically important flavonoids for enhanced production, but it might also be conceivable to impart improved plant resistance by manipulating the steps involved in phytoalexin synthesis, UV-protecting pigments (flavonols and anthocyanins), and better-aimed synthesis of *nod* gene influencers such as flavones and isoflavones [80,81]. It is well known that the flavonoid and anthocyanin pathways are the best-studied secondary metabolic pathways [82]. The antioxidant potential of flavonoids is being used to produce crops with increased stress resistance and to enhance the therapeutic potential of various crops.

Type III PKS superfamily of enzymes is the most important among the enzymes involved in secondary metabolic pathways. The active site of these homodimeric proteins iteratively participates in catalyzing the priming, extension, and cyclization reactions to produce a wide range of plant phenylpropanoids including flavonoids, chalcones, resveratrols, benzophenones, quinolones, phenylphenalenones, and curcuminoids [83]. Type III PKS enzymes fall into two main categories—namely, chalcone synthase (CHS) and non-chalcone synthase. Aldol type, lactonization type, no cyclization type, and nitrogen–carbon-forming type enzymes fall under the non-chalcone-synthesizing PKSs [84]. To study the role of active residues and to frame the structure in CHS and non-CHS, developing mutants is an ideal option. The mutants thus developed are capable of accepting bulkier substrates and the transformed plants showed improved growth and disease resistance. Recently, various mutation and crystallization studies were carried out and revealed the diverse function and structure of type III PKSs [85,86].

Engineering of secondary metabolite pathway was initiated in *Petunia* mutant RL01, which served as a recipient of the *A1* gene of *Zea mays*, encoding dihydroquercetin 4-reductase, reducing dihydrokaempferol to provide intermediate for pelargonidin biosynthesis that develops orange flowers [87]. Later, the role of flavonoids in the plant–microbe interaction arsenal was proposed by [88]. Studies have been conducted on *Aegle marmelos* [89] and *Emblica officinalis* [90] to unveil the role of active site amino acid residues and the phenotypic changes in their mutants. The overexpression of the quinolone synthase gene in *Aegle marmelos* induced improved growth, chlorophyll content, and photosynthetic efficiency. The CHS overexpressed transgenic plants showed an increased accumulation of secondary metabolites, such as flavonoids, phenols, and alkaloids that are accredited to their higher tolerance to drought and salinity stress. A similar result was obtained with *E. officinalis* CHS mutants. Mutants of *E. officinalis* CHS developed by exchange of single amino acids (F215S and F265V) were found to be utilizing *p*-coumaroyl–CoA as well as *N*-methylanthraniloyl–CoA as substrates and yield active products such as naringenin, 4-hydroxy 1-methyl 2(H) quinolone, and 1,3-dihydroxy-*n*-methyl acridone. Along with this finding, the mutants showed improved growth and disease resistance.

Apart from the genes involved in phenylpropanoid biosynthesis, several other transcription factors (TF) regulate the expression of key enzymes. Those TFs can be the targets for the enhanced production of flavonoids. In Arabidopsis, MYB12 TF was found to be upregulating the chalcone synthase (CHS) and flavone synthase (FS) genes, thereby accumulating flavonoids [91].

Most of the plant metabolic pathway engineering to date have used the constitutive promoter to enhance and activate the expression of native genes or transgenes in plants regardless of the tissue or the organ. One such promoter widely used is CaMV 35S promoter from the cauliflower mosaic virus, owing to its stability and activity in several plant species [92]. One such successful case was the introduction of the Totivirus antifungal protein KP4 in maize, rendering them resistant against the corn smut pathogen, *Ustilago maydis* [93]. Apart from biotic stresses, enhanced tolerance to abiotic stresses such as salt stress can also be achieved by such genetic engineering approaches. The levels of osmoprotectants such as trehalose or glycine betaine were achieved by overexpressing the genes from the cytochrome oxidase, trehalose-6-phosphate phosphatase (TPP), or trehalose-6-phosphate synthase (TPS) pathways [94]. Moreover, specific plant expression vectors were also used for introducing transgenes or genes for synthetic antimicrobial peptides, which render the plants resistant to a broader range of pathogens. Expression vectors such as pCAMBIA_1300_ were used for introducing a biopeptide BP_100_, a synthetic and cationic α-helical undecapeptide that exhibited a high degree of antibacterial activity against economically important plant-pathogenic bacteria with very low toxicity levels. Although the mutants exhibited antimicrobial activity, the fitness of the plants was compromised [95].

## 6. Managing the Reactive Oxygen Species

Overexpression of *AtMYB12* in transgenic Arabidopsis enhanced the production of flavonoids and showed a significant increase in ABA, proline, peroxidase (POD), and superoxide dismutase (SOD) activities, thereby improving the salt and drought tolerance of the plants [96]. Significant reduction in H_2_O_2_ and malonaldehyde (MDA) was also observed in the above study, which also indicated that tissue damage and necrosis were prevented by reducing the ROS content since ROS (especially H_2_O_2_) is considered to play the main role in hypersensitive responses [97]. In addition, several transcription factors are the positive regulatory molecules in plant immunity by upregulating defense hormones. Salicylic acid (SA), which is a phenolic phytohormone that plays an important role in systemic acquired resistance and also in pathogen pattern recognition, can be upregulated by overexpressing the systemic acquired resistance deficient 1 (SARD1) TF [98]. This dramatically increased the overall SA content in the plant and led to the constitutive expression of the pathogenesis-related (PR) marker genes *PR1* and *PR2*.

**Table 1:** List of mutants developed, which induce constitutive expression of defense-related genes.

Mutant Type	Encoding Protein	Activity	References
Constitutive expression of potato aspartate proteases in Arabidopsis	Aspartic proteases	Induces expression of genes under JA and SA signaling pathways such as PDF1.2 and PR1.	[71]
Transgenic lines expressing pokeweed antiviral protein	Antiviral protein	Constitutive expression of isoforms of PR-I and II genes	[99]
At edr1 mutant	Putative MAP kinase	Priming of *PR1* and *BGL2* genes	[100]
Constitutive expression of *VSP1*	Vegetative storage protein	Increases anthocyanin accumulation, constitutive expression of defense genes such as *VSP*, *VSP2*, *Thi2.1*, *PDF1.2*, *CHI.B*	[101]
Overexpression of At *JMT*	S-Adenosyl L- Methionine Jasmonic acid carboxyl methyl transferase	Constitutive expression of JA-responsive genes such as *VSP* and *PDF1.2*	[102]
Constitutive expression of At *ERF-1*	Transcription factor	Activates *CHI* and *PDF1.2*	[103]
Knockout of At *ACD11* gene	Sphingosine transfer protein	PCD and defense-related proteins	[104,105]
Overexpression of At *WRKY70*	Transcription factor	Constitutive expression of SA-responsive genes	[106]
Suppression of At *WRKY70*	Transcription factor	Constitutive expression of JA-responsive *PR* genes	[106]
Ectopic expression of *OsWRKY11*	Transcription factor	Constitutive expression of pathogen /drought defense responsive genes	[107]
Constitutive expression of *AtPROPEP1* gene	Elicitor of defense-related genes	Constitutive transcription of *PDF1.2* and defense-related proteins	[108]
Transgenic combination of wheat *Lr34*res and *Lr34*sus alleles	ABCG type transporter	Constitutive expression of defense- and secondary metabolite-related genes	[109]
*EDS1*/*PAD4* over-expression	Lipase-like protein	R genes activation	[110]
Constitutive expression of Senescence associated gene *SAG101* in *Arabidopsis*	Lipase-like defense regulator	Basal defense activation, R-mediated resistance	[111]

## 7. Conclusions

Complex mechanisms that evolved in plants help them to defend against both single and combination of stresses. It is indeed important to understand the complex defense mechanisms and signaling pathways for minimizing the stress effects on growth and reproduction, thereby improving their life span. It is evident that the studies on plant immunity against various combinations of stresses have revealed the complex nature of the signaling cross-talk. The information is still limited, even though the research continuously evolves due to the astounding progress in high throughput sequencing and well-developed bioinformatics technologies for genomic studies, proteomics, metabolomics, and whole-transcriptome profiling. Recently, the studies demonstrated that ROS synthesis, hormone signaling, MAPK pathways, and various transcription factors (TFs) can act as positive or negative regulators of the plant immune responses upon reception of biotic and abiotic stress stimuli, and are having non-specific, crucial roles in eliciting an immune response. The interaction between hormone-responsive and secondary metabolite biosynthetic genes is yet to be elucidated, which will allow for developing novel strategies to withstand the combination of both biotic and abiotic stresses encountered in field conditions.

Therefore, it is very important to understand and accelerate the development of stress-resistant crops via precise genome editing techniques, such as transcription activator-like effector nucleases (TALENs) and clustered regulatory interspaced short palindromic repeats (CRISPR)/Cas9 systems, and to promote the flourishment of plant biotechnology for the sustenance and welfare of living beings.

## Glossary

Mutation	A permanent alteration of the nucleotides in the genome sequence of an organism resulting a change in the gene product or affecting the function of gene partially or completely.
Apoptosis	Programmed cell death due to biochemical and structural changes in the cell.
Autoimmunity	Reduction in the activity of negative regulators or constitutive expression of defense genes that manifest retardation in growth and spontaneous lesion formation.
Metabolism	All chemical processes occurring in the plant cell for maintaining the cellular activities and for survival.
PR genes	Pathogenesis related genes are involved in the production of proteins that defend the plant in the event of a pathogen infection.
Phytoalexin	Antimicrobial compounds produced by plant tissues in response to contact with a pathogen and specifically inhibits its growth.
Oxidative stress	Stress generated by the reactive oxygen species when the plant system fails to detoxify them with the antioxidant enzymes.
Lignification	Process of strengthening the cell wall in plants by the synthesis and deposition of lignin.
TF	Transcription factors are proteins that controls the rate of transcription of DNA to RNA.
SA	Salicylic acid is a phenolic phytohormone that have roles in plant growth, photosynthesis and also mediate the defense pathways during pathogen attack.
JA	Jasmonic acid is a lipid based plant hormone that functions in photosynthesis, reproduction and biotic stress.
ET	Ethylene is a gaseous plant hormone involved in fruit ripening, flower opening and also in leaf abscission.
ABA	Abscisic acid is a plant hormone that have functions in stomatal closure, seed and bud dormancy and also in defense against environmental stresses.
Phenylpropanoids	Diverse family of secondary metabolites derived from amino acids phenylalanine and tyrosine.
